# A multidisciplinary review of the policy, intellectual property rights, and international trade environment for access and affordability to essential cancer medications

**DOI:** 10.1186/s12992-019-0497-3

**Published:** 2019-09-18

**Authors:** Sangita M. Baxi, Reed Beall, Joshua Yang, Tim K. Mackey

**Affiliations:** 1 Pardee RAND Graduate School, Santa Monica, CA USA; 2 Community Health Sciences, University of Calgary, Calgary, Alberta Canada; 3 Department of Public Health, California State University, Fullerton, Fullerton, CA USA; 4 Department of Anesthesiology, University of California, San Diego School of Medicine, San Diego, CA USA; 5 WHO Collaborating Centre for Governance, Transparency and Accountability in the Pharmaceutical Sector, University of Toronto, Toronto, Canada; 6 Global Health Policy Institute, 8950 Villa La Jolla Drive, A124, San Diego, CA 92130 USA

**Keywords:** Model list of essential medicines, EML, Pharmaceuticals, Access to medicines, Cancer, Patent status, World Health Organization, International Agency for Research on Cancer, Model Cancer list

## Abstract

In 2015, the World Health Organization (WHO) Expert Committee approved the addition of 16 cancer medicines to the WHO Model List of Essential Medicines (EML), bringing the total number of cancer medicines on the list to 46. This change represented the first major revision to the EML oncology section in recent history and reinforces international recognition of the need to ensure access and affordability for cancer treatments. Importantly, many low and middle-income countries rely on the EML, as well as the children’s EML, as a guide to establish national formularies, and moreover use these lists as tools to negotiate medicine pricing. However, EML inclusion is only one component that impacts cancer treatment access. More specifically, factors such as intellectual property rights and international trade agreements can interact with EML inclusion, drug pricing, and accessibility. To better understand this dynamic, we conducted an interdisciplinary review of the patent status of EML cancer medicines compared to other EML noncommunicable disease medicines using the 17th, 18th, 19th, 20th, and 21st editions of the list. We also explored the interaction of intellectual property rights with the international trade regime and how trade agreements can and do impact cancer treatment access and affordability. Based on this analysis, we conclude that patent status is simply one factor in the complex international environment of health systems, IPR policies, and trade regimes and that aligning these oftentimes disparate interests will require shared global governance across the cancer care continuum.

## Background

In February 2016, the slogan “We can. I can.” kicked off World Cancer Day 2016, accompanied by a 3 year campaign aimed at improving access to cancer care [1]. A key message was the need for collective action to “Improve access to cancer care”, and calls for countries to have a National Cancer Control Plan (NCCP) that “should cover access to … supportive and palliative care, high-quality cancer medicines and effective cancer treatment modalities” while concomitantly addressing affordability [1]. These goals are especially relevant for certain regions (e.g. Africa, Asia, and Latin America and the Caribbean) where 62% of new cases and 71% of cancer deaths occurred in 2018 [2]. By 2040, 65% of the world’s new cancer cases and 76% of cancer deaths are predicted to occur in these regions [3].

In order to facilitate better access to essential care, the World Health Organization (WHO) recommends that countries establish a national essential medicines list (NEML). Essential medicines should be selected based on criteria including public health relevance, evidence on efficacy and safety, and comparative cost-effectiveness [4]. Importantly, “access” to essential medicines means that these life-saving commodities are intended to be available within the context of functioning health systems at all times in “adequate amounts...with assured quality and adequate information, and at a price the individual and the community can afford”, a challenge particularly acute for developing countries where many patients pay for medicines out-of-pocket [5]. To that end, the WHO established the Essential Medicines List (EML) in 1977, which serves as a guide of what essential medicines to include on national formularies [6].

Importantly, patent status is not an explicit criterion of the EML, though undoubtedly it is a factor in drug pricing and accessibility. In fact, a 2012 study found that all EML drugs for noncommunicable diseases (NCDs) at the time were not covered by patents (up to the 17th edition), suggesting that access to essential medicines may also be influenced by other factors, such as availability and production of generics, prescribing practices, and health system financing [7]. Relatedly, studies have found large variations in cancer drug pricing at the health system level, primarily due to factors such as geography and medication type, which may also interact with intellectual property rights (IPRs) [8].

The link between EML status and national formulary inclusion is also not always strong, often differing by country and cancer drug product. A 2016 study looked at accessibility of cancer medications, including the 2015 EML additions, and found a large variance of 0.7 to 95% inclusion on national formularies from 135 countries; a more recent study found between 2 and 92% concordance (i.e. the number of medicines in common) with cancer drugs on the EML and national formularies in 116 countries [9, 10]. Both of these studies are also consistent with an Institute of Medicine report on the EML; concordance between the 18th edition of the EML (30 cancer medicines) and national formularies of several countries for cancer medications was inconsistent, and even countries with a limited number of cancer medicines on their national formulary have at least one drug that is not on the WHO EML [11].

Despite the complexity of EML status, national formulary inclusion, patent status, and global policy, there are few studies that have evaluated all these factors together while also taking into account the potential impact of international trade regimes on access to essential cancer medicines. Hence, there is a need to assess the IPR status of essential cancer drugs, while also assessing how IPR status might be impacted by and interact with the macro IPR and trade policy environment. Critically, the interaction between the rise in NCDs in low and middle-income countries (LMICs) mediated by health behaviors and socioeconomic status has also been shown to be associated with increases in the consumption of tobacco, alcohol, sugar, and processed foods, which can be facilitated by free trade agreements [12–14]. Hence, international trade regimes can also function as a critical part of the risk framework for NCDs and cancer prevention and access to treatment [15]. In response, this review article seeks to fill this knowledge gap in order to better understand what specific governance, policy, IPR, and trade-related aspects of health impact essential cancer drug access.

## Main text

### Methodology

**Fig. 1:**
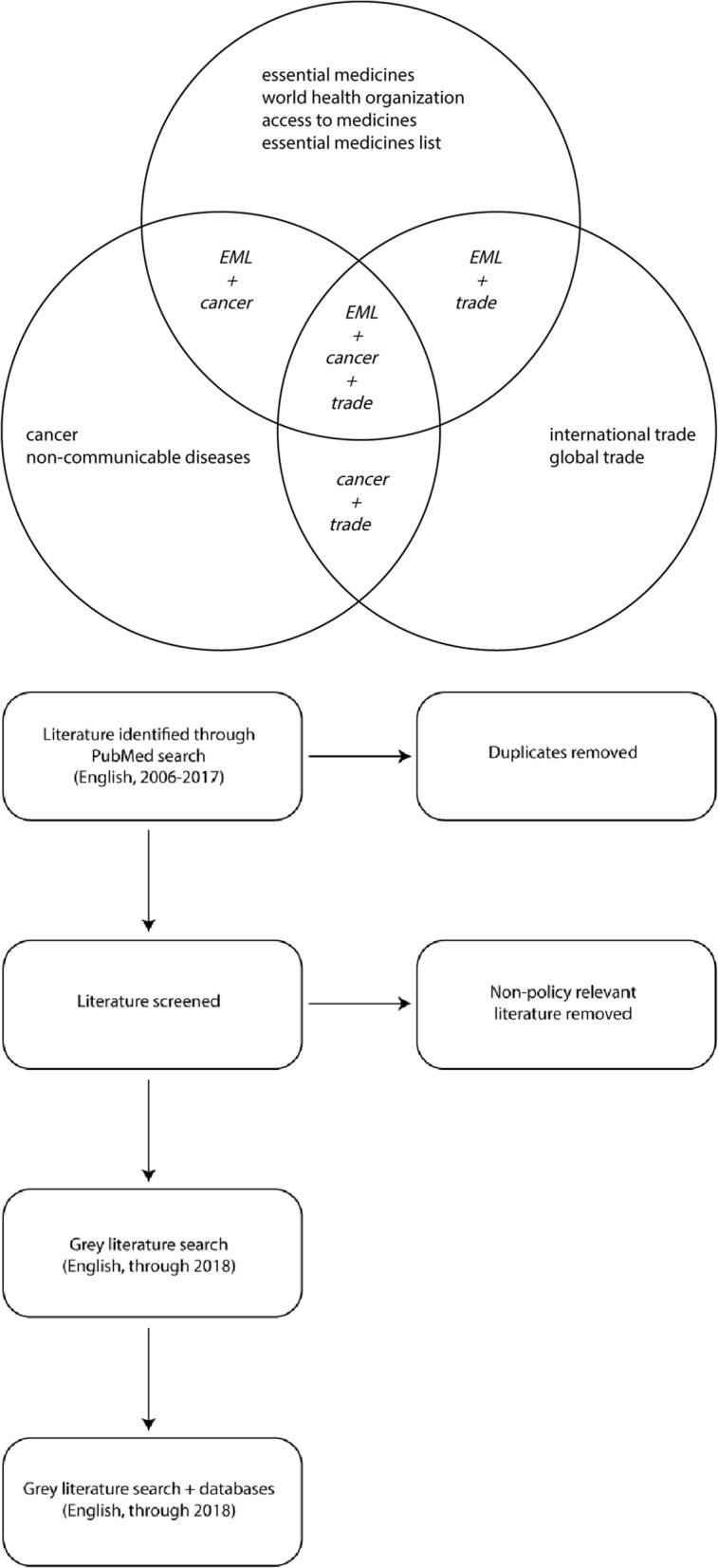
Search Strategy for Literature Review

In order to evaluate the governance, policy, IPR, and trade environment for essential cancer medicines, we conducted this study in two distinct phases: (1) literature review; and (2) secondary data analysis of patent status of WHO essential cancer medications. We first searched PubMed (Medline) for English-language articles published between 2006 and 2017, that contained the words “essential medicines”, “cancer”, “noncommunicable diseases”, “world health organization”, “international trade”, “global trade”, “access to medicines”, and “essential medicines list” in various combinations in the Title/Abstract field using the advanced search function as depicted in Fig. 1. We excluded literature reporting results for clinical studies, noncommunicable disease analyses that excluded cancer, and other literature that did not have policy-relevance to the topic. Additional sources of information included grey literature (informally published and generally non-peer reviewed written material) including technical reports/guidance from government agencies and multilateral organizations, and search results from government databases relevant to drug patent status. The goal of this literature search was to describe the history and process of cancer medication selection on the WHO EML, characterize the evolution of access and affordability to cancer drugs deemed to be essential by the WHO, and identify analysis related to the impact of international IPR and trade regimes on cancer drug access and affordability. The final reference list was generated on the basis of originality and relevance to the specific scope of this review.

In addition to our literature review, we conducted a descriptive IPR analysis for EML cancer medications to supplement information available in the literature. We conducted this secondary data analysis due to the lack of cancer IPR data in published articles and reports and with the aim of better quantifying patent characteristics for the sub-set of cancer drugs that are deemed essential. This also allowed us to compare patent status of essential cancer drugs to other essential NCD medications in order to assess whether there was a statistically significant different in the observed patent status characteristics between these two categories using a Pearson’s chi-squared test (*χ*^2^) in the programming language R in R Studio.

For our patent analysis, we examined the 17th, 18th, 19th, 20th, and 21st editions of the WHO EML and assessed the patent status of EML cancer medications compared to EML medicines used to treat other major NCDs using a framework adopted from a study conducted by *Mackey and Liang* 2012 in their patent analysis of NCD EML medications, which included cancer drugs [7]. We evaluated the sections of the EML and placed relevant treatments into four broad NCD categories: heart diseases & stroke, cancer, chronic lung diseases, and diabetes (Tables 2, 3, 4, and 5 in Appendix). For cancer, medicines for palliative care were also included in the analysis. Even if a medicine had multiple indications, it was only counted once per NCD category per edition.

The patent and exclusivity status in the U.S. for each medicine and its EML appropriate formulation(s) and dosage(s) were assessed by reviewing its status in the U.S. Food and Drug Administration Orange Book: Approved Drug Products with Therapeutic Equivalence Evaluations; biological formulations were assessed in Drugs@FDA: FDA Approved Drug Products, the Purple Book: Lists of Licensed Biological Products with Reference Product Exclusivity and Biosimilarity or Interchangeability Evaluations, and in the literature as required [16–21]. U.S. orphan drug designation and approval status were also evaluated using the U.S. FDA Orphan Drug Product Designation Database [16–21]. Formulation(s), dosage(s), and indication(s) were matched to the values provided on each EML.

We note that one limitation of this study was assessing patent status based on U.S. regulatory approval and exclusivity. Because the United States represents the world’s largest pharmaceutical market with a medicine patent registry, many patent assessment studies use U.S. patent status as an estimate of whether it may be patented elsewhere. While patent protection is granted on a country-by-country basis (often via a Patent Cooperation Treaty application) and only a fraction of products patented in the US are also patented in LMICs, many emerging markets (such as India and China) nevertheless patent these products as they are major centers for generic drug exports. We also note that this study did not assess certain applicant characteristics (e.g. geographic location, company size, revenue of company, etc.), though this should be explored in future studies. Please see Table 1 for a summary of the data obtained from each source.

**Table 1:** Data Sources

Data Source	Selection criteria based on listing on EML	Information Collected
Orange Book (FDA)	Generic drug name, formulation and dose, approved indication	Patent and exclusivity information
Purple Book (FDA)		Reference Product Exclusivity Expiry Date (if available), Biosimilar approval; else, search in the literature
Orphan Drug Database (FDA)		If the drug has an orphan designation (“Designated”) and if it received “Approval” to market as an orphan drug

## Results

### WHO essential medicines list and cancer

The EML undergoes evaluation every 2 years, and was most recently revised in March 2019 with the 21st and 7th editions of the EML and the Children’s EML (an EML specifying doses and formulations for pediatric populations), respectively [4, 22, 23]. Since its inception, the number of unique EML drugs has grown from 204 in 1977 to 460 drugs in 2019 [6, 22, 24]. While historically much of this growth is attributed to inclusion of anti-retrovirals for treatment of HIV/AIDS, more recent revisions have prioritized noncommunicable disease (NCD) medicines. This includes cancer, with the number of oncology products increasing from only seven in 1977 to 49 in 2017, with 16, 4, and 5 novel cytotoxic and adjuvant drugs added in the 2015, 2017, and 2019 revisions, respectively [24, 25]. With the inclusion of anti-hormone and palliative care therapies, there are now over 80 drugs on the EML recommended for cancer treatment regimens [24].

During revision of the EML, each subsection is reviewed by experts in their respective fields. For the 2015 revision (19th edition), WHO worked directly with the Union for International Cancer Control (UICC) to convene an expert working group to identify which cancer types had the potential for maximal treatment impact and to recommend treatments for inclusion prior to the EML revision [26, 27]. This work started at the beginning of 2014 and culminated with the recommendation of 52 cancer medicines to the WHO Expert Committee on the Selection and Use of Essential Medicines. In April 2015, the WHO Expert Committee met and added 16 new cancer medications to the 19th edition (and nine to the 5th edition of the Children’s EML) [27, 28]. It has been recommended that a similar protocol be followed for future EML revisions for cancer medicines; however, the two targeted cancer drugs added in the 20th edition in 2017 were included as alternatives for imatinib resistance [8, 24, 25, 27]. The most recent edition added 5 new targeted oncology products, including several drugs still covered by patents in the U.S.

Importantly, cancer treatment often involves multiple interventions, including surgery, radiotherapy, chemotherapy, targeted therapy, and palliative care, among others. This can equate to care that is costly both financially, as well as in terms of disability-adjusted life-years accounting for a worldwide loss of 169.3 million years of healthy life years and an economic loss of $895 billion in 2008 [29, 30]. In the US alone, the actual cost of cancer care in 2010 was estimated to be $124.57 billion [31]. Part of this estimated cost includes the expense of cancer medications, which can be impacted by IPRs, with growing attention to rising prices of cancer drugs a topic of public debate in the United States and other countries [32–34].

### Patent status of EML Cancer drugs

**Fig. 2:**
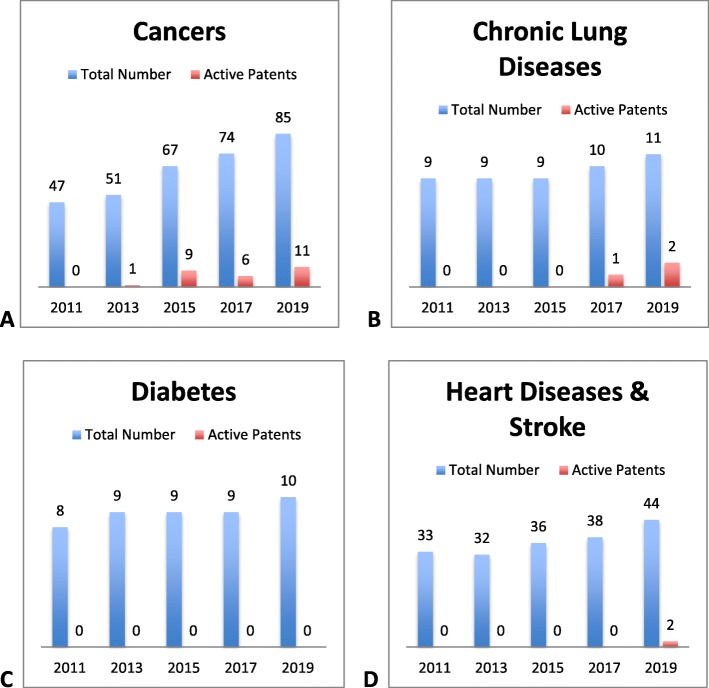
Patented medicines for noncommunicable diseases on the WHO EML, 2011-2019. The number above each bar reflects either the total number of medicines or the number of medicines with active medicines on the EML for the year listed

Our patent analysis of essential cancer medications examined five most recent editions of the WHO EML and assessed the patent status of EML cancer medications compared to EML medicines used to treat three other broad major NCD categories (Tables 2, 3, 4 and 5 in Appendix) [22, 24, 25, 35, 36]. For each edition, the number of EML medicines (regardless of patent status) for chronic lung diseases (*n* = 9, 9, 9, 10, 11, respectively), diabetes (*n* = 8, 9, 9, 9, 10), and heart diseases and stroke (*n* = 33, 32, 36, 38, 44) did not experience a substantial increase or decrease, and up to three patented medicines for these indications were identified in the last three EMLs (Fig. 2b, c, d) [25, 35, 36]. In fact, two newer medicines in the heart disease and stroke group, clopidogrel (anti-platelet agent) and enoxaparin (injectable low molecular-weight heparin), were added only after patent expiration. Enoxparin and clopidogrel lost patent exclusivity in the U.S. in 2008 and 2012, respectively, and both medicines were added to the EML in 2015 [17, 25, 36]. Within each disease, we examined the trend between time and the proportion of patented drugs; the results from Pearson’s *χ*^2^ test suggest that these factors are associated with patented drug inclusion over time for cancer indications (*p* = 0.0248), but not for chronic lung diseases (*p* = 0.2699), diabetes (*p* = 0.9876), or heart diseases and stroke (*p* = 0.5251).

In contrast, as previously discussed, the number of cancer medicines experienced a large increase in EML inclusion from 30 to 46 in 2015 for the 19th edition (excluding medicines for palliative care) [27]. The 16 new cancer medicines added to the EML in 2015 include: all-trans retinoic acid (ATRA), bendamustine, capecitabine, cisplatin, fludarabine, filgrastim, gemcitabine, imatinib, irinotecan, oxaliplatin, rituximab, trastuzumab, vinorelbine, anastrozole, bicalutamide, and leuprorelin. Half (*n* = 8) of these medicines were still covered by active patents in the U.S. at the time of the expert committee meeting to finalize the 19th EML (April 2015) [28].

**Fig. 3:**
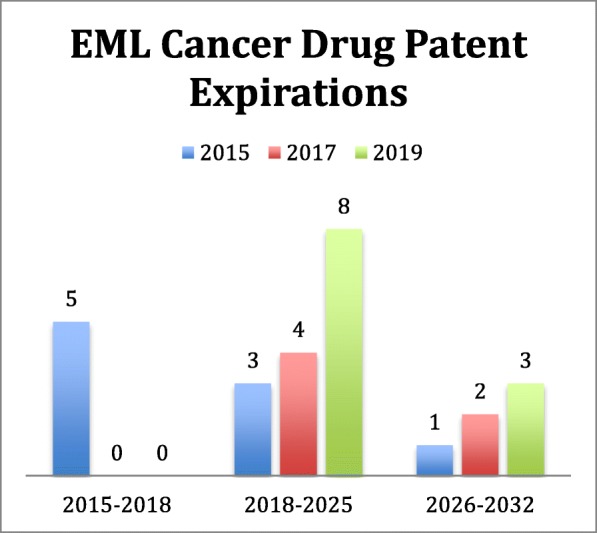
Expiry Dates of Patented Cancer Drugs on the 19th, 20th, and 21st Editions of the WHO EML (2015–2019)

In 2017, four new cancer drugs (along with two previous ones) were added to the EML; the two targeted therapies that were included are still under U.S. patent protection and are recommended for use in imatinib-resistant cancers [37]. Another five cancer drugs were added to the EML in 2019. In comparison, only one patented medicine for cancer was added in the 2013 EML revision (Fig. 2a, Table 3 in Appendix). We also evaluated the duration of patent coverage on each cancer medicine in the U.S. after its inclusion on the 18th, 19th, 20th, or 21st EML (Fig. 3). Of the patented cancer medicines currently on the EML, eight expire by the end of 2025, and the remaining patented medicines will expire in 2032 (*n* = 3) [16–18, 24, 25, 36].

With respect to competition for these essential cancer medicines, marketing by one or more generic or biosimilar manufacturers has now begun in the United States for 7 of the drugs (bendamustine, bevacizumab, capecitabine, imatinib, leuprorelin, oxaliplatin, fludarabine). In the case of fludarabine, the tablet formulation was voluntarily withdrawn by the manufacturer (Sanofi Aventis) in 2011, but marketing had begun for generic versions of the injectable formulation [38]. The FDA recently approved a generic or biosimilar version of dasatinib and trastuzumab, but marketing has not yet begun according to the National Drug Code Registry. No generic or biosimilar versions of nilotinib and rituximab have been approved for sale in the United States.

In 1983, the Orphan Drug Act (ODA) was passed to stimulate investment in rare disease therapy (~ 200,000 or less persons in the U.S.); one of the main benefits of the ODA is the additional 7 years of market exclusivity granted for the indication if the FDA approves the indication and the firm is the first to be approved [39]. However, this increased period of market exclusivity can also lead to higher prices, including for many cancer indications that fall under the rare disease or orphan designation [40]. In order to account for this characteristic, we also evaluated each cancer drug to see if it had been granted an orphan drug designation or approval sometime in the past for the same indication, formulation, and dose as its listing on the EML. In 21st edition of the list, of the 85 drugs that met our definition of cancer and palliative drugs, approximately 26 had an orphan designation, of which 20 were granted market exclusivity at approval. However, only 6 were still covered by a patent.

### International trade agreements, IPRs, and essential medicines

Another key factor that directly impacts access and affordability to essential cancer medications is the interaction between IPRs and the international trade regime and IPR provisions in trade agreements. International trade agreements established under the World Trade Organization (WTO) were designed to establish a rules-based system to reduce barriers to trade. The Trade-Related Intellectual Property Rights, or TRIPS Agreement, aims to harmonize, protect, and enforce IPRs and outlines minimum requirements for Member States to protect intellectual property, including 20-year minimum patent protection from filing [41].

Principally, TRIPS operates to enable IPR creation by providing incentives and harmonized protection for innovation, a design that also has the potential to impede access to essential medicines by granting product and market exclusivity. In 2001, the WTO adopted the “Declaration on the TRIPS Agreement and Public Health” at its 4th Ministerial Conference in Doha and later in 2003, the Paragraph 6 decision. Both serve to clarify rules around public health flexibilities and compulsory licenses (CLs) allowed under TRIPS (which allows a government authority to license the use of an invention to a third party or government agency without consent of the patent holders), so called “TRIPS flexibilities”, and were a direct response to activism around lack of access and high-costs for HIV/AIDS treatment [42]. Following negotiations in 2015, a waiver for the transitional period for applying pharmaceutical product IPRs under TRIPS was provided to least-developing countries, extending until 2033.

In an attempt to circumvent TRIPS flexibilities, there has been a trend in bilateral and multilateral free trade agreements (FTAs) that use TRIPS as a minimum standard but also push for stronger IPR protections, so-called “TRIPS-plus” measures [43, 44]. TRIPS-plus provisions are negotiated between individual countries and vary by FTA, but can include provisions that extend the term of a patent, eliminate TRIPS transitional periods, introduce barriers to or denial of compulsory licensing, and can restrict parallel importation (importing drugs purchased legally in another country for less than the local price). Other provisions negotiated in TRIPS plus FTA provisions include “evergreening” (e.g. acquiring new patents for a drug for minor changes without therapeutic improvement), restricting early working exceptions, requiring patent linkage mechanisms, granting patent protection for “new use”, restricting ability to interpret patentability criteria, and data exclusivity provisions (e.g. restricting access to innovator pre-clinical and clinical trial information for generic drug approval) [44].

Recent negotiations of “next generation” trade agreements have also provided important insights into the future of TRIPS-plus measures. These include provisions in the Trans-Pacific Partnership Agreement (TPPA) (TPPA never entered into force due to withdrawal of the U.S. but certain provisions were incorporated into the 2018 Comprehensive and Progressive Agreement for Trans-Pacific Partnership) for secondary patents, patent term extensions, data exclusivity, patent linkage provisions, and broader concerns about countries pushing for investor-state dispute settlement in the context of legal disputes for pharmaceuticals [45, 46].

For example, data exclusivity can create monopolies for expensive biologic cancer drugs because of the inherent difficulty in replicating the manufacturing process to establish bioequivalence for regulatory approval [47]. Commentators citing the American experience with free trade deals note that drug prices haven’t fallen in the US with Americans spending almost 100 billion dollars more for pharmaceutical products than 10 years ago [48]. Together, unfavorable IPR provisions in next generation trade agreements such as the suspended TPPA and the Transatlantic Trade and Investment Partnership (TTIP) have raised serious concerns from NGOs and public health advocates about the lack of safeguards in international trade to protect sustainable access to medicines.

### Policy and governance options

Though the WHO uses the EML to encourage individual states to rationally select medicines on the basis of comparative effectiveness, safety, and cost-effectiveness, EML status and the rationale for selection is but one factor that impacts access to these essential health products. For example, our IPR analysis indicates that few patented cancer drugs were on previous editions of the EML (Fig. 2a), despite guidelines that specify that patent status on its own should not bar a medicine from EML inclusion if all other requirements are met. However, patent status was reportedly openly considered as a factor in medicines inclusion decisions on the 19th EML.

Decision-making for the 19th edition of the EML (which led to the largest increase in inclusion for essential cancer medications observed) included over 90 oncology experts who assembled a recommendation list of cancer drugs aligned around the NCD Global Action Plan 2025 target of 80% availability of essential medicines and technologies. The published EML process openly discusses using patent status as a proxy indicator for cost as part of the decision-making process and also suggests that WHO EML inclusion can improve affordability [27, 49]. This position was somewhat clarified by the WHO EML Cancer Working Group for 2018–2019; they released both a report of their meeting as well as a thoughtful explanation of how they prioritize cancer medicines for inclusion in the EML [50, 51]. The group has an efficacy metric that they will use for each drug and indication that requests approval: a four to 6 month gain in overall survival. And while the working group stated that cost doesn’t come into inclusion decisions, they also emphasize that a therapy must “meaningfully prolong life” for inclusion on the EML.

When considering cost-effectiveness, it is important to note that patent status is simply one indicator that can impact access and affordability, as health and drug procurement policies (such as price caps and health financing/reimbursement models) can also influence drug pricing. If patent status is used as a decision-making factor, or a proxy indicator for cost, the guidelines should be updated and supported by evidence that tests the association between pricing and IPRs specific to cancer drug access that is also regional, country, health system, and coverage type specific. In fact, if the evidence supports IPRs as a key factor in affordability, current patent status and expiration should arguably be added as a formal weighted factor for EML inclusion. Additionally, though EML inclusion is intended to increase the availability and affordability of the medicines listed on a given national formulary, inclusion alone cannot address all the challenges and treatment complexity faced by cancer, which includes the need for diagnosis, medication, surgery, and other health interventions and their related financing.

In response, several measures to increase accessibility and affordability to NCD medicines have been discussed and proposed, including those drawing on experiences from the access to medicines movement for HIV/AIDS and other infectious diseases. Strategies include modifying licensing of medical product innovations and patents (e.g. socially responsible licensing, voluntary licensing, non-assert declarations), pooled purchasing to lower procurement/acquisition price, a proposed international treaty for biomedical research and development to enable medicines to become public goods, intellectual property/regulatory/market reform, utilizing health technology assessments for price-setting and reimbursement, moving to value-based pricing reimbursement schemes, among others [40].

In the context of the intersection between IPRs and trade, the first recommendation in the 2016 United Nations Secretary-General’s (UNSG) High-Level Panel on Access to Medicines report (a panel convened by former UN Secretary-General Ban Ki-moon to review and assess proposals to address policy incoherence between IPRs, human rights, trade, and public health for access to health technologies) was for states to recognize and leverage existing TRIPS flexibilities [52]. States appear to be exercising this option, with a 2018 study identifying 144 TRIPS flexibilities uses by over 80 countries between 2001 and 2016 [53]. The majority (81.8%) of these uses involved compulsory or public noncommercial use licenses, though of all the instances examined only 6.8% (*n* = 12), were used for cancer medications [53].

Importantly, to continue to ensure that TRIPS flexibilities can be appropriately leveraged, state governments need to consider their international trade landscape, including assessing the impact of IPR provisions in existing FTAs, while also assessing how to appropriately negotiate future FTAs so they do not include TRIPS-plus provisions. Pragmatically, states engaged in bilateral or multilateral trade negotiations must take into account the overall economic benefits of trade, but should also exercise caution in negotiations so they don’t barter away their existing rights under TRIPS and the Doha Declaration by introducing IPR provisions that act as barriers to the needs of their public health systems and populations.

Beyond avoiding TRIPS-plus provisions in FTAs, greater transparency, ensuring adequate representation of public health stakeholders, and broader public input by all negotiating countries is needed in international trade negotiations. Technical assistance and model language for FTAs that prioritizes public health interests, such as those available from the WHO, should form the basis for future bilateral and multilateral trade negotiations (including for the TPPA and its successor agreement the Comprehensive and Progressive Agreement for Trans-Pacific Partnership and TTIP.) Language in FTAs should explicitly reference, acknowledge and adhere to TRIPS flexibilities and establish these rights as controlling language over other agreements/provisions/chapters. Alternatively, these agreements should include explicit carve out language from trade provisions for pharmaceuticals similar to that negotiated for tobacco control measures during the TPPA [44]. States can also incorporate TRIPS flexibilities into national patent laws/legislation to ensure better compliance.

At the national level, there also needs to be concerted efforts to lower the acquisition and direct-patient cost of medicines. For example, a recent study of select oncology drugs in Thailand found national savings for certain cancer drugs on the NELM via compulsory licenses and price negotiation; for non-NELM cancer drugs, individual patient savings were obtained by patient assistance programs and marketing promotions [54]. Additional mechanisms to keep prices closer to the public procurement prices include improved supply chain governance; pooled procurement; government-run, non-profit pharmacies [55]; as well as more efficient supply chain management. Voluntary means can also be explored; Pfizer Inc. and Cipla Inc. have signed individual contracts with the American Cancer Society and the Clinton Health Access Initiative to provide 16 essential cancer medicines at competitive pricing in Ethiopia, Nigeria, Kenya, Uganda, Rwanda, and Tanzania [56]. This is a new initiative, and the pricing strategy is not fully known; however, pricing will need to be aggressive in order to secure accessibility in LMICs [57].

Finally, compulsory licenses are still a viable policy mechanism available under existing TRIPS flexibilities (though special considerations may apply with biologics and biosimilars) that have the power to potentially compel price negotiations between drug manufacturers or directly lower costs of drugs in LMICs [42, 58]. A recent analysis found that 10 years after the Doha Declaration, the majority of CLs and CL-associated activity occurred between 2003 and 2005 for HIV/AIDS medications, but of the few CL-associated episodes for NCDs, most were for cancer drugs in upper middle income countries [42]. Hence, cancer may represent a new frontier for CLs and those states actively seeking to exercise TRIPS flexibility rights in response to rising global cancer burden.

## Conclusions

The most recent revisions of the EML have made a concerted effort to add the best medicines possible for cancer treatment. In this decision-making, patent status appears to have also been a factor in the calculus for EML inclusion. As more cancer targeted therapies lose patent exclusivity, it will be important to assess whether generic and biosimilar versions become more accessible and affordable, and whether they are eventually included on the EML. However, patent status is simply one factor in a complex global environment of different health systems, IPR regimes, and trade agreements that impact access to and affordability of essential cancer medicines. Unifying global efforts to ensure that oftentimes diverging objectives of IPRs, trade, and public health are appropriately aligned will require shared governance viewing combating cancer as a key international economic, social, and human development issue.

This “shared” governance for cancer could be activated under the framework of the UN Sustainable Development Goals, with the ultimate aim of improving access to cancer medicines but also enabling cost-effective and equitable interventions across the cancer care continuum. Indeed, the framework for NCDs, including cancer, has already been laid out. NCDs have been recognized as one of the major challenges facing the Agenda for Sustainable Development. One of the key areas of anticipated implementation plans for the Agenda revolves around affordable access to new and existing therapies and also combating NCDs like cancer [59]. One needs look no farther than SDG Goal 3 targets 3.4 (“reduce by one third premature mortality from non-communicable diseases through prevention and treatment”), 3.8 ( “… access to safe, effective, quality and affordable essential medicines”), and finally 3.B ( “… provide access to affordable essential medicines and vaccines, in accordance with the Doha Declaration on the TRIPS Agreement and Public Health”), which reaffirms the right of countries to fully leverage TRIPS flexibilities to protect public health and enhance access to medicines.

The combined weight of these SDG targets and their associated indicators means that “We Can” align policy and governance of the EML, NELMs, IPRs, and trade agreements to ensure progress in global cancer prevention and treatment now and beyond the 2030 Agenda.

## Data Availability

Datasets were generated from publicly available sources including the World Health Organization and the United States Food and Drug Administration (please see links in references). The datasets supporting the conclusions of this article are included within the article (and the Appendix).

## Appendix

**Table 2:** Medicines for Heart Diseases & Stroke Included for Analysis

2011 EML	2013 EML	2015 EML	2017 EML	2019 EML	Treatment	2011 Patent	2013 Patent	2015 Patent	2017 Patent	2019 Patent
Y	Y	Y	Y	Y	acetylsalicylic acid	N	N	N	N	N
N	N	N	N	Y	alteplase	N	N	N	N	N
Y	Y	Y	Y	Y	amiloride	N	N	N	N	N
Y	Y	Y	Y	Y	amiodarone	N	N	N	N	N
Y	Y	Y	Y	Y	amlodipine	N	N	N	N	N
Y	Y	Y	Y	Y	bisoprolol	N	N	N	N	N
N	N	Y	Y	Y	clopidogrel	Y	N	N	N	N
N	N	N	N	Y	dabigatran	Y	Y	Y	Y	Y
Y	Y	Y	Y	Y	deferoxamine	N	N	N	N	N
N	N	Y	Y	Y	desmopressin	N	N	N	N	N
Y	Y	Y	Y	Y	digoxin	N	N	N	N	N
Y	Y	Y	Y	Y	dopamine	N	N	N	N	N
Y	Y	Y	Y	Y	enalapril	N	N	N	N	N
N	N	Y	Y	Y	enoxaparin	N	N	N	N	N
Y	Y	Y	Y	Y	epinephrine (adrenaline)	N	N	N	N	N
N	N	N	Y	Y	erythropoiesis-stimulating agents	Y	Y	Y	N	N
Y	Y	Y	Y	Y	ferrous salt	N	N	N	N	N
Y	Y	Y	Y	Y	ferrous salt + folic acid	N	N	N	N	N
Y	Y	Y	Y	Y	folic acid	N	N	N	N	N
Y	Y	Y	Y	Y	furosemide	N	N	N	N	N
Y	Y	Y	Y	Y	glyceryl trinitrate	N	N	N	N	N
Y	Y	Y	Y	Y	heparin sodium	N	N	N	N	N
Y	N	Y	Y	Y	hydralazine	N	N	N	N	N
Y	Y	Y	Y	Y	hydrochlorothiazide	N	N	N	N	N
Y	Y	Y	Y	Y	hydroxocobalamin	N	N	N	N	N
Y	Y	Y	Y	Y	hydroxycarbamide	N	N	N	N	N
Y	Y	Y	Y	Y	isosorbide dinitrate	N	N	N	N	N
Y	Y	Y	Y	Y	lidocaine	N	N	N	N	N
N	N	N	N	Y	lisinopril + amlopidine	N	N	N	N	N
N	N	N	N	Y	lisinopril + hydrochlorothiazide	N	N	N	N	N
N	N	N	Y	Y	losartan	Y	N	N	N	N
Y	Y	Y	Y	Y	mannitol	N	N	N	N	N
Y	Y	Y	Y	Y	methyldopa	N	N	N	N	N
Y	Y	Y	Y	Y	phytomenadione	N	N	N	N	N
Y	Y	Y	Y	Y	protamine sulfate	N	N	N	N	N
Y	Y	Y	Y	Y	simvastatin	N	N	N	N	N
Y	Y	Y	Y	Y	sodium nitroprusside	N	N	N	N	N
Y	Y	Y	Y	Y	spironolactone	N	N	N	N	N
Y	Y	Y	Y	Y	streptokinase	N	N	N	N	N
N	N	N	N	Y	telmisartan + amlopidine	Y	Y	N	N	N
N	N	N	N	Y	telmisartan + hydrochlorothiazide	Y	Y	Y	Y	Y
Y	Y	Y	Y	Y	tranexamic acid	N	N	N	N	N
Y	Y	Y	Y	Y	verapamil	N	N	N	N	N
Y	Y	Y	Y	Y	warfarin	N	N	N	N	N

*Y* included, *N* not included

**Table 3:** Medicines for Cancers, Hormones, and Palliative Care Included for Analysis

2011 EML	2013 EML	2015 EML	2017 EML	2019 EML	Treatment	2011 Patent	2013 Patent	2015 Patent	2017 Patent	2019 Patent
N	N	N	N	Y	abiraterone	Y	Y	Y	Y	N
Y	Y	Y	Y	Y	acetylsalicylic acid	N	N	N	N	N
N	N	N	N	Y	adalimumab	Y	Y	Y	Y	Y
N	N	Y	Y	Y	all-trans retinoid acid (ATRA)	N	N	N	N	N
Y	Y	Y	Y	Y	allopurinol	N	N	N	N	N
Y	Y	Y	Y	Y	amitriptyline	N	N	N	N	N
N	N	Y	Y	Y	anastrozole	N	N	N	N	N
N	N	N	N	Y	arsenic trioxide	Y	Y	Y	Y	N
Y	Y	Y	Y	Y	asparaginase	N	N	N	N	N
Y	Y	Y	Y	Y	azathioprine	N	N	N	N	N
N	N	Y	Y	Y	bendamustine	Y	Y	Y	Y	Y
N	Y	Y	Y	Y	bevacizumab	Y	Y	Y	Y	Y
Y	Y	Y	Y	Y	bicalutamide	N	N	N	N	N
Y	Y	Y	Y	Y	bleomycin	N	N	N	N	N
N	N	N	N	Y	bortezomib	Y	Y	Y	Y	Y
Y	Y	Y	Y	Y	calcium folinate	N	N	N	N	N
N	N	Y	Y	Y	capecitabine	Y	Y	Y	N	N
Y	Y	Y	Y	Y	carboplatin	N	N	N	N	N
Y	Y	Y	Y	Y	chlorambucil	N	N	N	N	N
Y	Y	Y	Y	Y	ciclosporin	N	N	N	N	N
N	N	Y	Y	Y	cisplatin	N	N	N	N	N
Y	Y	Y	Y	Y	codeine	N	N	N	N	N
Y	Y	Y	Y	Y	cyclizine	N	N	N	N	N
Y	Y	Y	Y	Y	cyclophosphamide	N	N	N	N	N
Y	Y	Y	Y	Y	cytarabine	N	N	N	N	N
Y	Y	Y	Y	Y	dacarbazine	N	N	N	N	N
Y	Y	Y	Y	Y	dactinomycin	N	N	N	N	N
N	N	N	Y	Y	dasatinib	Y	Y	Y	Y	Y
Y	Y	Y	Y	Y	daunorubicin	N	N	N	N	N
N	Y	Y	Y	Y	dexamethasone	N	N	N	N	N
Y	N	N	Y	Y	diazepam	N	N	N	N	N
Y	Y	Y	Y	Y	docetaxel	N	N	N	N	N
Y	Y	Y	Y	Y	docusate sodium	N	N	N	N	N
Y	Y	Y	Y	Y	doxorubicin	N	N	N	N	N
N	N	N	N	Y	erlotinib	Y	Y	Y	Y	Y
Y	Y	Y	Y	Y	etoposide	N	N	N	N	N
N	N	N	Y	Y	fentanyl	N	N	N	N	N
N	N	Y	Y	Y	filgrastim	Y	Y	N	N	N
N	N	Y	Y	Y	fludarabine - injection	N	N	N	N	N
N	N	Y	Y	Y	fludarabine - tablet	Y	Y	Y	Y	N
Y	Y	Y	Y	Y	fluorouracil	N	N	N	N	N
Y	Y	Y	Y	Y	fluoxetine	N	N	N	N	N
N	N	Y	Y	Y	gemcitabine	N	N	N	N	N
N	Y	Y	Y	Y	haloperidol	N	N	N	N	N
Y	Y	Y	Y	Y	hydrocortisone	N	N	N	N	N
Y	Y	Y	Y	Y	hydroxycarbamide	N	N	N	N	N
Y	Y	Y	Y	Y	hyoscine butylbromide	N	N	N	N	N
N	N	N	Y	Y	hyoscine hydrobromide	N	N	N	N	N
Y	Y	Y	Y	Y	ibuprofen	N	N	N	N	N
Y	Y	Y	Y	Y	ifosfamide	N	N	N	N	N
N	N	Y	Y	Y	imatinib	Y	Y	Y	N	N
N	N	Y	Y	Y	irinotecan	N	N	N	N	N
Y	Y	Y	Y	Y	lactulose	N	N	N	N	N
N	N	N	N	Y	lenalidomide	Y	Y	Y	Y	Y
N	N	Y	Y	Y	leuprorelin (leuprolide acetate)	Y	Y	Y	N	N
N	Y	Y	Y	Y	loperamide	N	N	N	N	N
N	N	N	N	Y	melphalan	N	N	N	N	N
Y	Y	Y	Y	Y	mercaptopurine	N	N	N	N	N
Y	Y	Y	Y	Y	mesna	N	N	N	N	N
N	N	N	Y	Y	methadone	N	N	N	N	N
Y	Y	Y	Y	Y	methotrexate	N	N	N	N	N
Y	Y	Y	Y	Y	methylprednisolone	N	N	N	N	N
N	Y	Y	Y	Y	metoclopramide	N	N	N	N	N
Y	Y	Y	Y	Y	midazolam	N	N	N	N	N
Y	Y	Y	Y	Y	morphine	N	N	N	N	N
N	N	N	Y	Y	nilotinib	Y	Y	Y	Y	Y
N	N	N	N	Y	nivolumab	–	–	Y	Y	Y
Y	Y	Y	Y	Y	ondansetron	N	N	N	N	N
N	N	Y	Y	Y	oxaliplatin	Y	Y	Y	N	N
Y	Y	Y	Y	Y	paclitaxel	N	N	N	N	N
Y	Y	Y	Y	Y	paracetamol	N	N	N	N	N
N	N	N	N	Y	pegaspargase	N	N	N	N	N
Y	Y	Y	Y	Y	prednisolone	N	N	N	N	N
Y	Y	Y	Y	Y	procarbazine	N	N	N	N	N
N	N	N	N	Y	realgar-Indigo naturalis formulation	N	N	N	N	N
N	N	Y	Y	Y	rituximab	Y	Y	Y	N	N
Y	Y	Y	Y	Y	senna	N	N	N	N	N
Y	Y	Y	Y	Y	tamoxifen	N	N	N	N	N
N	N	N	N	Y	thalidomide	Y	Y	Y	Y	Y
Y	Y	Y	Y	Y	tioguanine	N	N	N	N	N
N	N	Y	Y	Y	trastuzumab	Y	Y	Y	Y	Y
Y	Y	Y	Y	Y	vinblastine	N	N	N	N	N
Y	Y	Y	Y	Y	vincristine	N	N	N	N	N
N	N	Y	Y	Y	vinorelbine	N	N	N	N	N
N	N	N	Y	Y	zoledronic acid	Y	Y	N	N	N

*Y* included, *N* not included

**Table 4:** Medicines for Chronic Lung Diseases Included for Analysis

2011 EML	2013 EML	2015 EML	2017 EML	2019 EML	Treatment	2011 Patent	2013 Patent	2015 Patent	2017 Patent	2019 Patent
Y	Y	Y	Y	Y	beclometasone	N	N	N	N	N
Y	Y	Y	Y	Y	budesonide	N	N	N	N	N
N	N	N	Y	Y	budesonide + formoterol	–	Y	Y	Y	Y
Y	N	N	N	N	chlorphenamine	N	N	N	N	N
Y	Y	Y	Y	Y	dexamethasone	N	N	N	N	N
Y	Y	Y	Y	Y	epinephrine (adrenaline)	N	N	N	N	N
Y	Y	Y	Y	Y	hydrocortisone	N	N	N	N	N
Y	Y	Y	Y	Y	ipratropium bromide	N	N	N	N	N
N	Y	Y	Y	Y	loratadine	N	N	N	N	N
Y	Y	Y	Y	Y	prednisolone	N	N	N	N	N
Y	Y	Y	Y	Y	salbutamol	N	N	N	N	N
N	N	N	N	Y	tiotropium	Y	Y	Y	Y	Y

*Y* included, *N* not included

**Table 5:** Medicines for Diabetes Included for Analysis

2011 EML	2013 EML	2015 EML	2017 EML	2019 EML	Treatment	2011 Patent	2013 Patent	2015 Patent	2017 Patent	2019 Patent
Y	Y	Y	Y	Y	acetazolamide	N	N	N	N	N
N	N	N	N	Y	diazoxide	N	N	N	N	N
Y	N	N	N	N	glibenclamide	N	N	N	N	N
N	Y	Y	Y	Y	gliclazide	N	N	N	N	N
Y	Y	Y	Y	Y	glucagon	N	N	N	N	N
Y	Y	Y	Y	Y	insulin injection (soluble)	N	N	N	N	N
Y	Y	Y	Y	Y	intermediate-acting insulin	N	N	N	N	N
N	Y	Y	Y	Y	latanoprost	Y	N	N	N	N
Y	Y	Y	Y	Y	metformin	N	N	N	N	N
Y	Y	Y	Y	Y	pilocarpine	N	N	N	N	N
Y	Y	Y	Y	Y	timolol	N	N	N	N	N

*Y* included, *N* not included

## Abbreviations

AIDS: Acquired immune deficiency syndrome
CL: Compulsory license
EML: Essential Medicines List
FDA: U.S. Food and Drug Administration
FTA: Free trade agreement
HIV: Human immunodeficiency virus
IPR: Intellectual property rights
LMICs: Low and middle-income countries
NCCP: National Cancer Control Plan
NCD: Noncommunicable disease
NEML: National Essential Medicines List
TPPA: Trans-Pacific Partnership Agreement
TRIPS: Trade-Related Intellectual Property Rights
UNSG: United Nations Secretary-General
WHO: World Health Organization
WTO: World Trade Organization
